# Single-cell transcriptomics profiling reveals cellular origins and molecular drivers underlying melanoma brain metastasis

**DOI:** 10.1371/journal.pone.0336502

**Published:** 2025-11-24

**Authors:** Jingxin Zeng, Ling Lin, Yangyang Ma, Wenzhe Huang, Quan Luo, Ju Wen

**Affiliations:** 1 The Affiliated Guangdong Second Provincial General Hospital of Jinan University, Guangzhou, Guangdong, China; 2 Guangzhou Dermatology Hospital, Guangzhou, Guangdong, China; 3 The Air Force Hospital of the Southern Theater Command, Guangzhou, Guangdong, China; 4 Department of Biomedical Engineering, General Hospital of Eastern Theater Command Hospital, Nanjing, Jiangsu, China; Karl-Franzens-Universitat Graz, AUSTRIA

## Abstract

Melanoma brain metastasis (MBM) remains lethal with limited treatment efficacy. Meanwhile, the cellular origins and drivers of brain metastasis from melanoma have yet to be defined. Through integrated single-cell/single-nucleus RNA sequencing of 26 melanoma samples, we identified a pre-brain-metastatic tumor subpopulation (MBMATCs, MBM-associated tumor cells) within a conserved malignant cell trajectory (Mela3). MBMATCs exhibited activated pro-metastatic pathways and upregulated neural/adhesion genes (*NRG3*, *NCAM1*), suggesting a cellular origin for brain tropism. MBM ecosystems showed T cell exhaustion (elevated *PD-1*, *HAVCR2*, *LAG3*) and Treg enrichment. An MBM-Index derived from bulk RNA-seq accurately quantifies MBMATCs abundance, independently predicting poor overall survival in both TCGA-SKCM and validation cohorts. Furthermore, we assessed the clinical relevance of the MBM-Index and uncovered five candidate drugs with potential activity against MBMATCs. This study identifies MBMATCs as brain metastasis associated tumor cells and positions the MBM-Index as a biomarker for early stratification of melanoma patients at high risk of brain metastasis.

## Introduction

Melanoma, a highly aggressive form of skin cancer, accounts for over 75% of annual skin cancer-related deaths, posing a significant global health challenge [1]. The disease originates from genetic mutations in pigment-producing melanocytes, leading to uncontrolled cell proliferation and invasion [2,3]. Its aggressive growth patterns and high metastatic potential have escalated melanoma into a major public health concern worldwide. According to the World Health Organization (WHO) classification, the principal subtypes of melanoma includes superficial spreading melanoma, nodular melanoma, lentigo malignant melanoma, and acral melanoma (AM) to exhibit distinct clinical and histopathological features [4]. While AM has a relatively low global incidence, it constitutes approximately 50% of melanoma cases in East Asian populations, highlighting the importance of understanding subtype-specific disease characteristics [5].

The propensity of melanoma to disseminate early during tumor progression significantly impacts patient outcomes. Epidemiological studies reveal that around 44% of metastatic melanoma patients develop melanoma brain metastasis (MBM), a devastating complication associated with profound morbidity and mortality [2]. Once MBM occurs, the median survival plummets to a mere 4−5 months, reflecting the disease’s rapid progression and limited treatment efficacy [2,6,7]. The unique physiological barriers of the brain, particularly the blood-brain barrier (BBB), severely restrict the delivery of many chemotherapeutic agents, rendering traditional systemic therapies largely ineffective. Even with the advent of targeted therapies, such as combinations of BRAF and MEK inhibitors (BRAFi/MEKi), which have increased the intracranial response rate in MBM patients from 25−40% (with BRAF inhibitors alone) to 58%, the durability of this response remains significantly shorter compared to extracranial disease [8–15]. Furthermore, immune checkpoint inhibitors (ICIs), particularly anti-CTLA-4 (ipilimumab) and anti-PD-1 (nivolumab, pembrolizumab) antibodies, either alone or in combination, have demonstrated durable intracranial responses and have become a standard of care for MBM [12]. landmark studies such as CheckMate 204 and NIBIT-M2 have shown that combination ipilimumab and nivolumab can yield intracranial clinical benefit rates exceeding 50%, with a subset of patients achieving long-term survival [11,16]. However, a significant proportion of patients still exhibit primary resistance or develop secondary resistance to ICIs, underscoring the complexity of the brain immune microenvironment and the urgent need for reliable predictive biomarkers [17,18]. This not only exacerbates the physical suffering of patients but also leads to substantial psychological distress for patients and their families, burdening healthcare systems with costly, end-stage interventions.

Metastatic melanoma progression is characterized by phenotypic switching toward slowly proliferating but highly invasive cellular states [19]. The microphthalmia-associated transcription factor (MITF) serves as a master regulator of melanogenesis and melanocyte development. Elevated MITF expression promotes localized proliferation while suppressing invasion, inflammation, and epithelial-to-mesenchymal transition (EMT) [17]. Consequently, MITF^high^ melanoma cells exhibit a proliferative yet non-invasive phenotype, whereas MITF^low^ populations demonstrate enhanced invasiveness and metastatic potential [18,20–22] This phenotypic plasticity is further mediated by AXL-driven molecular reprogramming, which facilitates the transition from an epithelial to an aggressive mesenchymal state in melanoma [23]. Mounting evidence underscores the distinct molecular and biological characteristics that set MBM apart from metastases in other organs. RNA sequencing studies have revealed differential expression of key genes involved in angiogenesis, immune evasion, and cell adhesion, highlighting the unique tumor-microenvironment interactions in the brain [24]. For instance, tumors with enriched immune gene and signaling network expression are associated with improved survival in MBM patients, suggesting that the immune landscape plays a crucial role in brain metastasis progression. Moreover, specific genetic mutations, such as those in BRAF, NRAS, or the phosphatidylinositol 3-kinase pathway, have been linked to an elevated risk of brain metastasis [6]. Recent research has also identified stress-induced HDAC8 activity as a regulator of an invasive melanoma cell state that promotes MBM development, further emphasizing the complex molecular mechanisms underlying this process [25]. Although the critical roles of MITF and AXL in melanoma invasion and metastasis have been established, whether they exhibit distinct roles in brain metastasis remains unclear.

Despite advancements in drug delivery strategies aimed at overcoming the BBB and improving treatment efficacy, current approaches for MBM remain inadequate. The urgent need for reliable predictive indicators for brain metastasis cannot be overstated.

In this study, we leverage transcriptomic data analysis to identify specific gene expression patterns and biomarkers that can serve as predictive indicators for melanoma brain metastasis, aiming to contribute to a more comprehensive understanding of MBM biology and the development of more effective clinical management strategies.

## Materials and methods

### Data collection of melanoma PT, ECM and MBM samples

The single-cell RNA sequencing (scRNA) of 6 primary melanoma (PT) samples were obtained from GSE215121 (GSM6622292–GSM6622294, GSM6622299–GSM6622301) [5]. The single-nucleus RNA sequencing (snRNA) of 10 ECMs (extracranial melanoma metastases) and 10 MBMs (melanoma brain metastases) samples were collected from GSE185386 (GSM6022256- GSM6022265 and GSM6022273- GSM6022282) [26]. According to the original publication (Biermann et al., *Cell* 2022), these ECM samples were derived from subcutaneous metastases (n = 9) and an axillary lymph node metastasis (n = 1).

Additionally, bulk transcriptomic data and clinical annotations for melanoma patients were retrieved from public repositories, including The Cancer Genome Atlas (TCGA, https://portal.gdc.cancer.gov/) and the Gene Expression Omnibus (GEO). This dataset comprised a melanoma brain metastasis modeling cohort (TCGA, n = 473) and two independent validation cohorts (GSE190113, n = 101; GSE65904, n = 210). Following exclusion of samples designated “normal” those with missing survival data, or those with an overall survival (OS) duration <30 days, 308 TCGA-SKCM samples were retained for downstream analysis.

### Single-cell RNA sequencing data processing and cell annotation

Single-cell/nucleus RNA sequencing (sc/snRNA-seq) data from 26 melanoma patients across two cohorts were integrated into a unified Seurat object (v5.2.1) [27]. Quality control thresholds excluded cells with the following criteria: mitochondrial gene content exceeding 20%, and cells with either fewer than 500 or more than 5,000 expressed genes.

### Doublets were identified and removed using DoubletFinder (v2.0.4)

Filtered gene-barcode matrices were normalized using the ‘LogNormalize’ method. The top 2,000 highly variable genes were identified via the ‘vst’ method in the ‘FindVariableFeatures’ function. Gene expression matrices were subsequently scaled and centered using the ‘ScaleData’ function. Principal component analysis (PCA) was performed, and the top 40 principal components were selected for downstream t-Distributed Stochastic Neighbor Embedding (t-SNE) dimension reduction. Other parameters were kept at their default settings. We employed Harmony integration with parameters (theta = 2, sigma = 0.1, lambda = 1) specifically tuned to aggressively correct for batch effects arising from both technological sources (scRNA-seq vs. snRNA-seq) and sample biological origins. Cell clusters were resolved at a resolution of 0.3 (FindClusters), followed by t-SNE for nonlinear dimensionality reduction (RunTSNE). Cell identities were annotated based on canonical marker expression profiles. Unless specified, all functions used default parameters.

### Ro/e (Ratio of observed to expected) analysis

The Ratio of observed to expected (Ro/e) was calculated to identify cell types enriched in specific tissues (PT, ECM, MBM) [28]. For a given cell type and tissue, the Ro/e value represents the ratio of the observed proportion of that cell type in the tissue to the expected proportion. A value greater than 1 indicates enrichment in that specific tissue.

### Scoring of T Cell Functional States

Gene signatures for Naive, Activation/Effector function, Exhaustion, and Cytotoxicity were obtained from Wang et al. [29]. Single-cell signature scores were computed using the AddModuleScore algorithm in Seurat.

### Pseudotime trajectory analysis

Cellular state transitions were reconstructed using Monocle (v2.34.0) [30]. Differentially expressed genes (DEGs) were identified per cluster via the differentialGeneTest function (q-value < 1 × 10 ⁻ ⁵) and employed to order cells along pseudotime. Dimensionality reduction was performed with reduceDimension (DDRTree method), and trajectories were visualized in two-dimensional space via plot_cell_trajectory, annotated by pseudotime values. The Branched Expression Analysis Modeling (BEAM) approach was employed to identify regulatory genes at branch point 2, thereby deriving genes governing the metastasis of primary tumor cells to ECM and MBM.

### Copy number variation (CNV) estimation

Tumor cell copy number variations (CNVs) were inferred from single-cell RNA sequencing (scRNA-seq) data using InferCNV (v1.22.0). The Seurat-derived count matrix served as input, with high-quality T cells and macrophages providing non-malignant reference baselines for CNV inference.

### Gene set variation analysis (GSVA)

Hallmark pathway enrichment within individual cells of the Melanoma Mela3 subgroup was quantified using the GSVA package (v2.0.6) in R, with gene sets curated from the Molecular Signatures Database (MSigDB; https://www.gsea-msigdb.org/gsea/msigdb) [31]. Differential pathway activity between cell groups was assessed via enrichment score comparison using the limma package [32]. Hallmark pathways exhibiting |t-value| > 42 (false discovery rate < 0.05) were defined as the most significantly enriched.

### Deciphering transcriptional programs underlying tumor cell intrinsic heterogeneity

We applied the GeneNMF R package (v0.7.0) to implement the NMF algorithm on tumor cells, identifying eight MPs (metastatic programs). After excluding low-silhouette-scoring MP3 and MP8, six universally expressed programs in melanoma tumors were retained. Using the AddModuleScore function in Seurat, we assessed the enrichment of these programs in each tumor cell based on program-specific signatures.

### Cell-cell communication analysis

Cell-cell communication networks were inferred from the integrated single-cell/single-nucleus RNA sequencing (sc/snRNA-seq) data using the CellChat R package (v2.1.2). The analysis leveraged the canonical ligand-receptor pairs from the CellChatDB.human database to compute communication probabilities

### MBM-Index quantification in bulk RNA-seq data

Raw count data from the transcriptomic profiles were first normalized to Transcripts Per Million (TPM) to account for sequencing depth and gene length variations. The MBMATC-specific gene set was used as the training feature matrix. A one-class logistic regression (OCLR) model, implemented via the “gelnet” R package, was applied to extract the transcriptional signature representative of the MBMATC state [33]. The MBM-Index for each patient in the TCGA-SKCM cohort (n = 308) was computed by performing Spearman correlation analysis between the weighted MBMATC signature and the normalized expression matrix of each sample. The correlation coefficients were then linearly scaled to a range of 0–1 to generate the final MBM-Index score. A higher index value indicates a stronger resemblance of the tumor’s transcriptome to the MBMATC signature, thereby reflecting a greater risk of brain metastasis.

### Therapeutic drug screening for MBMATCs and high MBM-Index patients

Drug sensitivity predictions for MBMATC-enriched and high MBM-Index cohorts were generated using the oncoPredict R package (v1.2). Gene expression profiles from the Cancer Cell Line Encyclopedia (CCLE) were integrated with drug response data from the Cancer Therapeutics Response Portal (CTRP) and GDSC2 database [34,35]. The core function, calcPhenotype, was applied to our transcriptomic data (including MBMATCs and patient samples stratified by MBM-Index) to compute a drug sensitivity score for each sample-drug pair, which is analogous to a predicted IC50 value. To identify drugs with selective efficacy against the high-risk populations, we implemented a two-tiered screening strategy: (1) For the MBMATC cellular state: We compared the sensitivity scores of MBMATC-enriched samples versus non-MBMATC samples. A drug was considered a candidate if it showed significantly greater potency in MBMATCs, defined by a log2 fold-change (log2FC) < −0.1 and a Mann-Whitney U test p-value < 0.05. (2) For the high MBM-Index patient cohort: A parallel comparison was performed between samples with a high MBM-Index versus those with a low index, using the same statistical thresholds (log2FC < −0.1; U test p < 0.05). The final candidate drugs were selected by taking the intersection of the hits identified from the CTRP and GDSC2 databases for each respective group.

### Wound healing assay

Melanoma cells were seeded in 6-well plates at a density of 5 × 10^5^ cells per well and cultured until full confluence. A single scratch wound was generated in each monolayer using a sterile 10 μL pipette tip. After washing with PBS to remove detached cells, the cells were incubated in fresh medium containing the indicated concentrations of Fluvastatin (10 μM and 30 μM). Scratch closure was monitored and imaged at 24h and 48h post-scratching using an inverted microscope.

### Statistical analysis

All analyses were conducted in R (v4.4.3). Inter-group differences were assessed using Wilcoxon rank-sum or Kruskal-Wallis tests. Associations between variables and survival were evaluated via univariate and multivariate Cox proportional hazards regression models, with Kaplan-Meier analysis and log-rank testing quantifying subtype-specific survival differences.

## Results

### The comparative landscape of primary melanomas, extracranial metastases, and brain metastases

**Fig 1:**
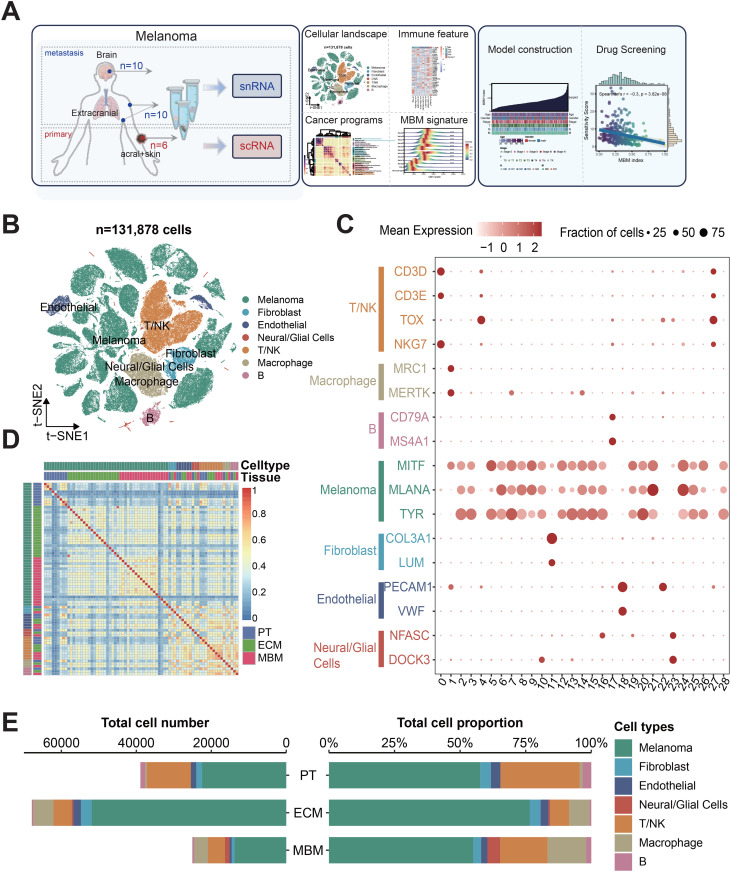
Single cell RNA-seq and snRNA-seq profiling of the PTs (primary melanoma), ECMs (extracranial melanoma metastases) and MBMs (melanoma brain metastases) environments. (A) Experimental workflow for integrative analysis of PT, ECM, and MBM microenvironments. (B) t-SNE projection annotating major cell types across PT, ECM, and MBM samples. (C) Dot plot depicting expression levels of canonical markers across 29 cell clusters. (D) Heatmap of Spearman’s correlations among each cell type for samples from different tissue origins. (E) Bar plot demonstrating the abundance and proportional distribution of major cell populations in PT, ECM, and MBM.

To elucidate the cellular dynamics in melanoma brain metastatic progression, we integrated sc/snRNA-seq data of 26 melanoma tumors across two cohorts, comprising 6 primary tumor (PTs), 10 extracranial melanoma metastases (ECMs) and 10 melanoma brain metastases (MBMs) (Fig 1A). Following strict quality control, 131,878 cells were retained for downstream analysis. Batch correction with Harmony effectively addressed variations across samples and technological sources, resulting in transcriptomes that clustered primarily by cell type identity rather than by technology or sample origin in the t-SNE visualization, confirming the robustness of the integrated dataset (Fig 1B and S1 Fig). Based on canonical marker genes for each major cell type, we annotated 29 clusters into seven distinct lineages: melanoma cells, fibroblasts, endothelial cells, Neural/Glial cells, T/NK cells, macrophages, and B cells (Fig 1B-C). Spearman correlation analysis revealed stronger inter-cellular-type heterogeneity than variations attributable to tissue origins, demonstrating the robustness of our annotation (Fig 1D). Immune cells exhibited higher intra-group similarity than malignant cells, while malignant cells partitioned into distinct submodules, highlighting their transcriptional heterogeneity. Analysis of tissue-specific cell type composition revealed significant differences in the tumor microenvironment (TME) (Fig 1E, S1 Table). Melanoma cells predominated in all sample types: PTs (57.6%), ECMs (76.5%), and MBMs (55.0%) (Fig 1E). Notably, T/NK cells constituted 30.1% of PTs but only 7.2% in ECMs and 18.1% in MBMs (Fig 1E). Conversely, macrophage proportions increased dramatically from 1.1% in PTs to 14.7% in MBMs, suggesting immune microenvironment remodeling during metastasis (Fig 1E).

### T cell-primed immunosuppressive microenvironment in melanoma brain metastases

**Fig 2:**
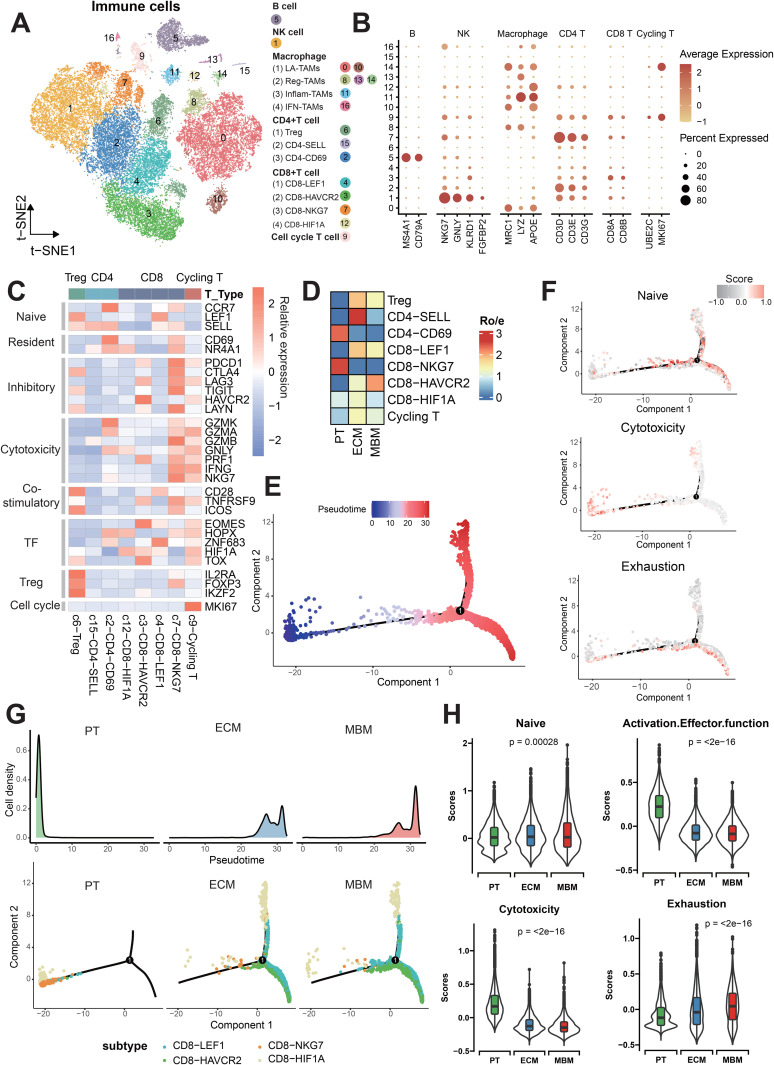
Immune landscape and CD8^+^ T cell transition states in PTs, ECMs and MBMs. (A) t-SNE projection of immune cells, annotated by cell clusters and major subsets. (B) Dot plot depicting percentage and mean expression of signature genes across 17 immune clusters. (C) Heatmap of gene module expression in T-cell subtypes. (D) Heatmap depicting tissue-specific Ro/e enrichment scores across eight T cell subtypes. (E) Pseudotemporal trajectory of CD8 ⁺ T cells across PTs, ECMs and MBMs. (F) Dynamics of naïve (top), cytotoxic (middle), and exhausted (bottom) gene signatures along pseudotime. (G) State distribution of pseudotime-ordered CD8 ⁺ T cells from PT (left), ECM (center), and MBM (right), with cell density curves showing at the top of the figure. (H) Violin plots comparing expression kinetics of signature genes across CD8 ⁺ T-cell states in PT, ECM, and MBM. Boxplot elements: center line (median), box bounds (IQR), whiskers (1.5 × IQR). Statistical significance assessed by Kruskal-Wallis test.

Melanoma exhibits unique sensitivity to immunotherapies, where the nature of immune infiltration (termed “hot” or “cold” microenvironment) predicts treatment response and clinical outcomes [36–38]. Significant differences were observed in immune cell profiles across PTs, ECMs and MBMs (S2A-C Fig). We isolated 32,828 immune cells for independent dimensionality reduction and clustering to characterize their functionally specialized subtypes. Based on lineage-specific marker expression, these cells were classified into six major types (Fig 2A-B). Macrophages were further subdivided into LA_TAMs, Reg_TAMs, Inflam_TAMs, and IFN_TAMs (S2D Fig); T cells comprised CD4^+^ T cells, CD8^+^ T cells, and Cycling T cells (S2E Fig). The Treg subset exhibited high expression of *IL2RA*, *FOXP3* and *IKZF2* (Fig 2C). Naive populations, including CD4-SELL and CD8-LEF1, enriched *CCR7*, *LEF1* and *SELL*. CD4-CD69 represented tissue-resident T cells, while the CD8-NKG7 subset demonstrated cytotoxic activity through effector molecules (*GZMK*, *GZMA*, *GNLY*, *IFNG*). Conversely, CD8-HAVCR2 cells displayed immunosuppressive functions, expressing checkpoint regulators (*PDCD1*, *LAG3*, *HAVCR2*). Notably, we identified a distinct Cycling T population predominantly expressing *MKI67* (Fig 2C). The CD8-HIF1A T cell subset, characterized by high expression of *HOPX*, *HIF1A*, and *NR4A1*, along with low expression of checkpoint genes, cytotoxic genes, and naive cell genes, likely constitutes a tolerance-adaptive T cell population induced by the hypoxic microenvironment that gains survival advantages at the expense of effector functions (Fig 2C). To quantify tissue-specific enrichment, we calculated the Ratio of observed to expected (Ro/e) for each T cell subset [28]. We clearly observed that CD4-CD69 and CD8-NKG7 T cell subsets were preferentially enriched in PT, whereas Tregs and CD8-HAVCR2 subsets showed a greater propensity for MBM tissues (Fig 2D). Collectively, these data demonstrate T cell-mediated immunosuppressive remodeling during brain metastasis.

### Transitional states of CD8 ⁺ T cells during melanoma metastasis

Pseudotemporal trajectory analysis via Monocle [39] reconstructed the dynamic progression of four CD8 ⁺ T cell subsets. Pseudotime trajectory analysis integrating T-cell functional signatures revealed a progressive shift from cytotoxic dominance to terminal exhaustion. Naive, cytotoxicity, and exhaustion signatures were scored and projected onto a unified pseudotime axis, demonstrating that cytotoxicity primarily drives trajectory initiation, while naive signatures mark transitional states, and exhaustion dominates the terminal phase (Fig 2E-F). Cell density mapping along this trajectory showed distinct spatial distributions: PTs clustered predominantly at the initiation point, ECMs occupied transitional and terminal regions, and MBMs concentrated almost exclusively at the terminal endpoint (Fig 2G). Subpopulation mapping further validated this progression: trajectories consistently originated from the cytotoxic CD8-NKG7 subset, transitioned through the CD8-LEF1 intermediate state, and terminated in the exhaustion-dominated CD8-HAVCR2 subset (Fig 2G). Notably, we identified CD8-HIF1A as a divergent terminal branch, suggesting an alternative T cell fate trajectory under hypoxic or aggressive microenvironmental pressures. Naive signature scores were marginally higher in ECM, whereas PT samples showed the highest scores in activation effector function and cytotoxicity. In contrast, MBM displayed the highest exhaustion scores compared to the other two groups (Fig 2H). These results demonstrate spatially divergent CD8 ⁺ T cell differentiation during metastasis, with MBMs exhibiting pronounced T cell exhaustion.

### Distinct transcriptomic programs in melanoma brain metastases

**Fig 3:**
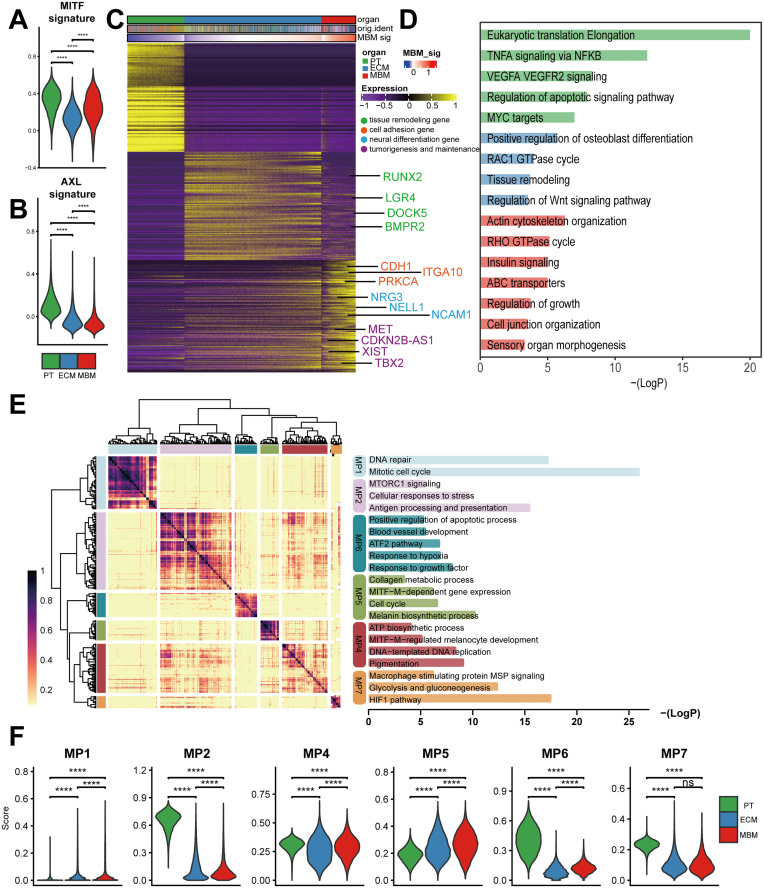
Tumor cell-specific transcriptomic features across PTs, ECMs and MBMs. (A and B) Distribution of MITF- and AXL-signature scores in PT, ECM, and MBM tumor cells. Wilcoxon rank-sum test. (C) Heatmap depicting expression of signature genes uniquely enriched in each tissue. (D) Enriched pathways for each tissue-specific gene set were performed using Metascape. (E) Heatmap showing the correlation of all 239 signatures determined from the cNMF algorithm, 6 highly correlated expression programs are highlighted. Top enriched pathways of 5 tumor expression programs (right panel). (F) Comparative activation scores of NMF-derived transcriptomic metaprograms across PTs, ECMs and MBMs, with statistical significance assessed via Wilcoxon rank-sum tests.

To characterize cancer cell signatures in MBMs, we compared the expression of two established programs: the AXL-high program (associated with invasiveness and drug resistance) and the MITF-high program (defined by melanocyte lineage markers including MITF) [26,40,41]. Consistent with the prior findings of Biermann et al. [42], we observed that MBMs showed higher MITF-high program scores than ECMs, while ECMs exhibited stronger AXL-high signature enrichment. Extending this analysis to primary tumors, we found that PTs displayed the highest scores for both programs (Fig 3A–B, S2 Table). Collectively, our results confirm and extend the observation by Biermann et al. that the AXL-high program is not a defining feature of MBMs, while further demonstrating its dynamic regulation across the full spectrum of melanoma progression, from primary tumors to extracranial and brain metastases.

To delineate the unique characteristics of tumor cells in MBMs, we performed differential gene expression analysis. This revealed that tumor cells in MBMs not only exhibited significantly upregulated tumorigenesis and maintenance genes (e.g., *MET* and *TBX2*), but also expressed neural differentiation markers (e.g., *NRG3*, *NELL1*, *NCAM1*, *ADNP*) and cell adhesion molecules (e.g., *CDH1*, *PRKCA*) (Fig 3C, S3 Table). In contrast, ECMs predominantly activated tissue remodeling genes, including *BMPR2*, *LGR4* and *DOCK5*. Gene enrichment analysis identified distinct pathway activation patterns between MBMs and ECMs: MBMs exhibited significant enrichment in actin cytoskeleton organization, RHO GTP cycle, insulin signaling and ABC transporters, whereas ECMs displayed overrepresentation of positive regulation of osteoblast differentiation, RAC1 GTPase cycle, tissue remodeling and WNT signaling (Fig 3D). Notably, PTs showed prominence in proliferative pathways (eukaryotic translation, MYC targets) and inflammatory responses (TNFα/VEGFα). Together, the progression from PT to ECM to MBM is accompanied by a shift in core pathways from “proliferation/angiogenesis” to “migration/metabolic adaptation”, underscoring microenvironment-driven phenotypic remodeling.

To delineate transcriptional programs underlying intra-tumoral malignant cell heterogeneity, we applied consensus non-negative matrix factorization (NMF) to identify predominant functional signatures and modules across all tumor cells. Hierarchical clustering consolidated 239 gene signatures into six transcriptomic metaprograms (MPs) (Fig 3E). MP1 was cell cycle-associated (e.g., *MCM4*, *WDHD1*, *BRCA1*), while MP2 encompassed immune and stress responses marked by MHC gene upregulation (*HLA-A*, *HLA-B*, *CTSB*, *CALR*). MP4 and MP5 exhibited striking similarities: MP4 was enriched in the regulation of pigmentation and melanocyte energy metabolism, while MP5 was characterized by the melanin biosynthetic process and cell cycle. MP6 was predominantly enriched in pathways related to growth and angiogenesis, while MP7 exhibited robust activation of HIF1-mediated hypoxia responses and glycolytic metabolic activity (Fig 3E). Program activity quantification showed MBMs had significantly elevated MP1 (cell cycle) and MP5 (melanin biosynthesis) scores versus PT/ECM (Fig 3F), implicating proliferation-pigmentation synergy in brain metastatic fitness.

### Pseudotemporal trajectory identifies MBM-associated tumor cell subpopulations

**Fig 4:**
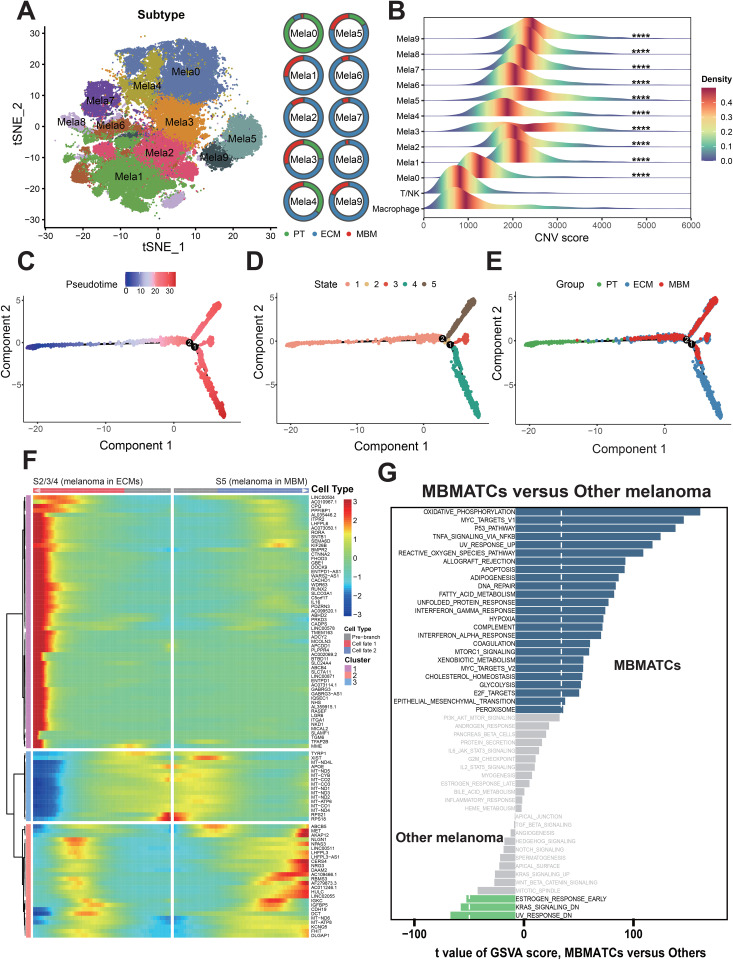
Identification of melanoma brain metastasis-associated tumor cell subpopulations (MBMATCs). (A) Melanoma tumor cell subtype classification from integrated scRNA-seq and snRNA-seq data. Circular plot depicts tissue origins across ten subtypes. (B) Copy number variation (CNV) scores in melanoma subtypes versus reference cells (Macrophage and T/NK cells). Significance levels: P < 0.05, P**** < 0.0001 (Wilcoxon rank-sum test). (C-E) Pseudotime trajectory analysis of Mela3 cells, ordered and annotated by pseudotime (left panel), five cellular states (middle panel) and three tissues (right panel). (F) Trajectory analysis of cluster Mela3 identified three cellular states at branch point 2, organized into one root state and two divergent branches resulting from cell fate decisions. Heatmap depicting 100 branch-dependent genes across three states defined by branched expression analysis modeling. White arrows indicate fate transitions from the pre-branch state (S1) to divergent fates: S2/3/4 (cell fate 1) or S5 (cell fate 2). (G) GSVA revealed differential activation of hallmark pathways in brain metastasis-associated tumor cells (MBMATCs) versus other tumor subpopulations within the Mela3 cluster.

To delineate tumor cell subpopulations implicated in melanoma brain metastasis (MBM), we classified 79,246 malignant tumor cells from primary tumors (PTs), extracranial metastases (ECMs), and MBMs into 10 transcriptionally distinct clusters (Mela 0–9) (Fig 4A). Tissue origin analysis revealed distinct metastatic patterns: Mela2, Mela6, Mela7, and Mela8 subtypes were predominantly derived from extracranial metastases (ECMs) (88%, 93%, 93%, and 96%, respectively), whereas Mela0 primarily originated from primary tumors (PTs) (90%) (S4 Table). Only the Mela3, Mela4, and Mela5 demonstrated substantial representation across all three tissue types. Specifically, Mela3 accounted for more than 25% of malignant cells in each group. Based on copy number variation (CNV) inference, the Mela3, Mela4, and Mela5 exhibited high CNV loads, ranking fourth, ninth and second, respectively, confirming their malignant nature (Fig 4B). To identify tumor cell states specifically associated with brain metastatic progression, we performed pseudotime analysis separately on the Mela3, Mela4, and Mela5. In both Mela4 and Mela5, no cellular state was found where MBM cells constituted over 50% of the state population, indicating a lack of strong MBM-specific differentiation bias (S3 Fig). In contrast, analysis of the Mela3 cluster revealed a clear and specific distribution of states across tissues (Fig 4C-E). State S5 was predominantly enriched in MBMs (67.2%), while States S2, S3, and S4 were collectively enriched in ECMs (88.4%). State S1 was the most ubiquitously distributed, present in PTs (76.1%), ECMs (14.1%), and MBMs (9.8%) (Fig 4D-E).

To pinpoint the key genes driving the differentiation from a PT-like state (S1) to the MBM-enriched state (S5), we applied Branched Expression Analysis Modeling (BEAM). This analysis identified 100 genes that critically regulate the branching tumor cell trajectories from State S1 towards State S5 (MBM) and the ECM-associated states (S2/S3/S4) (Fig 4F). Genes driving S5 differentiation exhibited dual signatures: neural regulation (e.g., *NRG3*, *CDH19*, *KCNQ5*, *DLGAP1*) and pro-metastatic signaling (e.g., *MET*, *AKAP12*, *IGFBP5*). Given that primary tumor (PT) cells in Mela3 possess latent potential for MBM transformation, we defined this subpopulation as MBMATCs (melanoma brain metastasis-associated tumor cells). Gene Set Variation Analysis (GSVA) revealed marked enrichment of pro-metastatic and proliferation pathways in melanoma brain metastasis-associated tumor cells (MBMATCs) versus primary tumor (PT) malignant cells (Fig 4G), suggesting a potential mechanism underlying the excellent brain metastatic competence of MBMATCs.

### Association of MBM-Index with clinicopathological features in TCGA-SKCM

To investigate the link between MBMATCs and the immunosuppressive microenvironment, we inferred cell-cell interactions from ligand-receptor signaling networks in single-cell RNA-seq data. We found that CD8-HAVCR2 in ECMs and MBMs received significantly more interaction signals than PTs (S4A Fig). Further analysis with CellChat revealed that in ECMs and MBMs, other CD8 ⁺ T cell subsets exhibited more frequent interactions with CD8-HAVCR2 via HLA class I molecules binding to CD8A/CD8B receptors (S4B Fig). Notably, specifically within MBM samples, the Mela3 subpopulation (MBMATCs) uniquely engaged CD8-HAVCR2 via LAMA4 and FN1 interacting with the CD44 receptor (S4B Fig). These signaling interactions suggest that MBMATCs may orchestrate immune evasion within the MBM microenvironment

**Fig 5:**
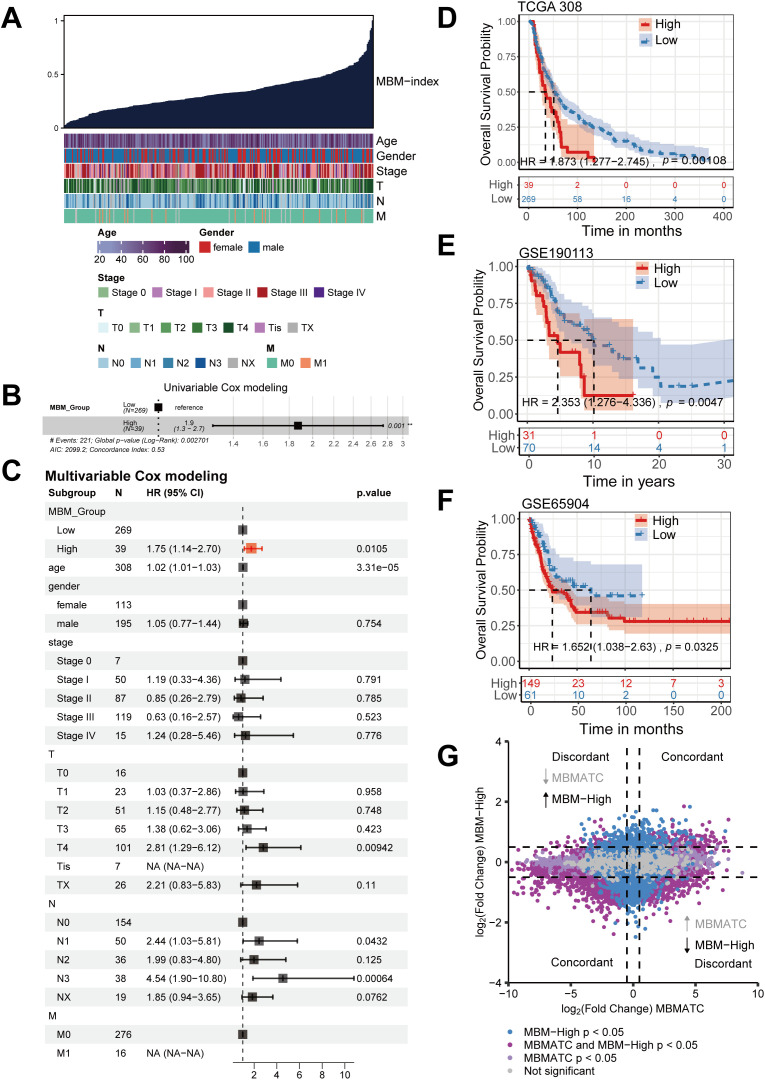
The MBM-Index as an independent predictor of overall survival (OS) in melanoma. (A) Association between MBM-Index and clinicopathological features. Columns indicate patients ranked by ascending MBM-Index (top row), with subsequent rows showing clinical attributes. (B) Univariable Cox proportional hazards model analysis of TCGA dataset samples (n = 308) using MBM-Index groups revealed significant prognostic utility for melanoma progression. Hazard ratios (HRs) and 95% confidence intervals (CIs) are presented as box-and-whisker plots. (C) Multivariable Cox regression demonstrating prognostic utility of MBM-Index independent of clinical covariates. Box plots display hazard ratios (HR) and 95% confidence intervals (CI). (D-F) Kaplan-Meier curves showing significantly reduced OS in high- versus low-MBM-Index groups across TCGA-SKCM (D), GSE190113 (E), and GSE65904 (F) datasets. (G) Scatter plot depicting the correlation of log₂ fold change values for all transcripts differentially expressed between MBMATCs and other tumor cells, and between MBM-High and MBM-Low groups. Colored dots indicate significant differential expression genes in the specified groups. T, N, M refer to the AJCC TNM staging system classification (Tumor size, Node involvement, distant Metastasis).

Given the critical role of MBMATCs in brain metastasis, we leveraged their distinct transcriptional signature to construct an MBM-Index using a one-class logistic regression algorithm. This index quantifies the brain metastasis risk for each sample in the bulk RNA-seq data from the 308 patient TCGA-SKCM cohort (Fig 5A). The MBM-Index showed no significant correlation with age, sex, tumor stage, or TNM classification (S5 Fig). Univariate Cox regression identified the MBM-Index as a prognostic factor for overall survival (OS) (Fig 5B). Multivariable analysis confirmed its independence from conventional clinicopathological variables (HR = 1.75, 95% CI 1.14–2.7, P = 0.0105; Fig 5C). Kaplan-Meier analysis revealed significantly reduced overall survival (OS) in patients with high MBM-Index (HR = 1.873, 95% CI 1.277–2.745, log-rank P = 0.001; Fig 5D). We independently validated this prognostic signature in two multicenter melanoma cohorts (GSE190113 and GSE65904). Based on bulk transcriptome data (RNA-seq or microarray), patients were stratified into high- or low-MBM-Index subtypes. Survival analysis confirmed shorter survival in the high-MBM-Index group versus the low-MBM-Index group (GSE190113: HR = 2.353, 95% CI 1.276–4.336, P = 0.005; GSE65904: HR = 1.652, 95% CI 1.038–2.63, P = 0.033; Fig 5E-F). Functional enrichment analysis of co-expressed genes in melanoma brain metastasis-associated tumor cells (MBMATCs) and high-MBM-Index samples revealed consistent activation of pro-metastatic pathways, including cell migration regulation, immune cytokine signaling, and axon guidance mechanisms (Fig 5G and S6 Fig). These findings indicate that elevated MBM-Index independently predicts brain metastasis risk and reduced survival in melanoma, beyond conventional staging systems.

### Identification of therapeutic compounds targeting MBMATCs in high MBM-Index patients

**Fig 6:**
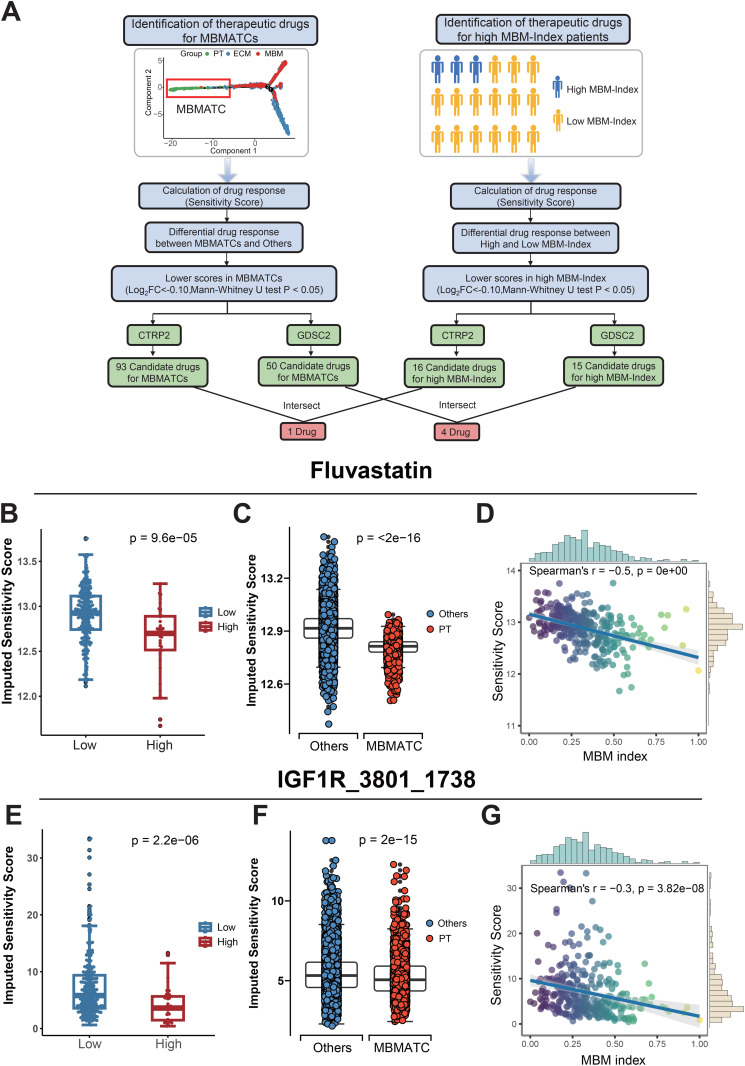
Discovery of therapeutic compounds targeting brain metastasis-associated tumor cells (MBMATCs) in high MBM-Index melanoma. (A) Dual-pathway screening strategy for identifying MBMATC-targeted compounds and high-MBM-Index patient-selective agents. (B-D) Differential drug-response analysis (boxplots) and Spearman’s correlation analysis (right panel) for Fluvastatin. (E-G) Differential drug-response analysis (boxplots) and Spearman’s correlation analysis (right panel) for IGF1R_3801_1738.

To identify therapeutic compounds with potential efficacy against melanoma brain metastasis, we employed a two-tiered computational drug screening strategy focusing on: (1) the MBM-associated tumor cell (MBMATC) transcriptional signature, and (2) the transcriptomic profile of patients with a high MBM-Index (Fig 6A). Drug sensitivity predictions were generated using the oncoPredictalgorithm, which integrates gene expression data from the Cancer Cell Line Encyclopedia (CCLE) with drug response profiles from the CTRP and GDSC2 databases.

Screening of the CTRP and GDSC2 compound libraries identified 93 and 50 candidates, respectively, that were predicted to be effective against the MBMATC signature. Parallel screening for the high MBM-Index patient cohort yielded 16 (CTRP) and 15 (GDSC2) candidate agents. The intersection of hits from both approaches revealed a convergent set of compounds: Fluvastatin (from CTRP analysis) and four candidates from the GDSC2 library (IGF1R_3801_1738, Mirin_1048, PF-4708671_1129, XAV939_1268) (Fig 6A). These selected compounds exhibited significantly lower predicted sensitivity scores (indicative of higher efficacy, analogous to a lower IC₅₀) in both MBMATCs and high MBM-Index patients compared to their counterparts. Furthermore, the sensitivity scores for these compounds demonstrated a significant negative correlation with the MBM-Index values across the patient cohort (Fig 6B–G; S7 Fig), suggesting that patients with a higher risk of brain metastasis may be more vulnerable to these therapeutic agents.

**Fig 7:**
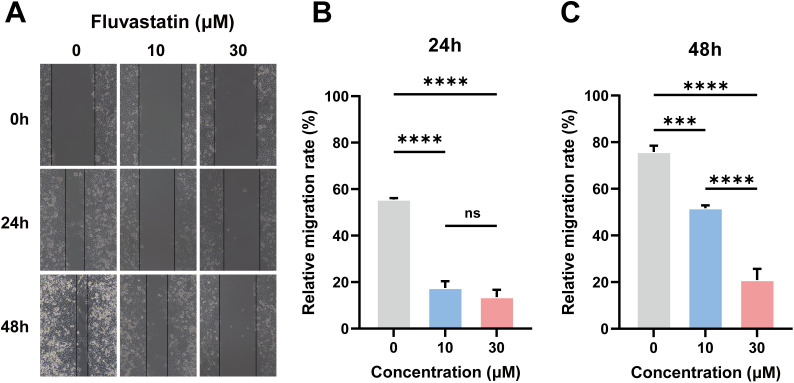
Fluvastatin inhibits the migration of melanoma A375 cells in a wound healing assay. (A) Representative microscopic images of cell scratches at 0, 24, and 48 hours after treatment with different concentrations of Fluvastatin. (B) Quantitative analysis of cell migration rate measured by wound closure area at 24h. (C) Quantitative analysis of cell migration rate measured by wound closure area at 48h. Data are presented as mean±SEM; statistical significance is indicated (ns, not significant; ***p < 0.001; ****p < 0.0001).

To experimentally validate the anti-metastatic potential of our top computational candidate, we performed an in vitro wound healing assay using A375 melanoma cells treated with Fluvastatin. The results demonstrated that Fluvastatin treatment (30 μM) significantly inhibited cell migration at both 24h and 48h time points compared to the control group (Fig 7A-C). Specifically, at the 48h, Fluvastatin led to a 72.43% reduction in wound closure (p < 0.0001), providing functional evidence that it effectively impairs the migratory capacity of melanoma cells (Fig 7C).

## Discussion

Melanoma brain metastasis (MBM) represents a devastating complication with limited therapeutic options and dismal survival outcomes. Despite advances in targeted and immunotherapies, the blood-brain barrier (BBB), intrinsic molecular heterogeneity, and immunosuppressive microenvironment continue to challenge effective MBM management. Our study leveraged sc/sn-RNA sequencing to dissect the cellular and molecular underpinnings of MBM progression, revealing key mechanisms driving brain tropism and immune evasion.

Consistent with studies highlighting the immunosuppressive nature of brain metastases across cancers [42–44], we observed profound T-cell exhaustion specifically within the MBM microenvironment. The pseudotemporal trajectory of CD8 ⁺ T cells demonstrated a transition from cytotoxic (CD8-NKG7) and hypoxia-adapted (CD8-HIF1A) states in primary tumors toward terminally exhausted (CD8-HAVCR2) states in MBMs. This spatial divergence in T-cell differentiation, characterized by elevated expression of *PD-1*, *TIM-3*, and *LAG3*, aligns with reports of T-cell dysfunction in central nervous system (CNS) malignancies [45] and underscores the rationale for combining checkpoint blockade with hypoxia-modulating therapies to reinvigorate antitumor immunity in MBM patients [46]. The significant enrichment of Tregs in MBMs further reinforces an immunosuppressive TME, potentially driven by CNS-specific immune tolerance mechanisms.

Contrary to the prevailing model associating AXL-high states with metastatic dissemination [47], our transcriptomic analysis revealed that MBMs exhibit higher MITF-high program activity than extracranial metastases (ECMs), while primary tumors (PTs) displayed the strongest AXL-high signatures. This suggests that the AXL-high invasive phenotype may be more critical for initial dissemination than for established brain colonization. Instead, MBMs uniquely upregulated genes involved in neural differentiation (*NRG3*, *NELL1*), cell adhesion (*NCAM1*), and cytoskeletal remodeling (RHO GTP cycle), indicating adaptive reprogramming for neuronal niche engagement [26].

A pivotal finding was the identification of melanoma brain metastasis-associated epithelial cells (MBMATCs) within the conserved Mela3 cluster. Pseudotime trajectory analysis positioned these cells at a branching point preceding metastatic commitment, exhibiting activation of pro-metastatic pathways (e.g., cell migration, cytokine signaling) distinct from other PT malignant cells. This mirrors the “brain metastasis-associated epithelial cells” (BMAECs) concept in LUAD [48], suggesting a conserved paradigm where rare, transcriptionally primed subclones within primary tumors drive brain tropism. Interestingly, cell-cell communication analysis revealed more frequent interactions between HLA class I molecules and the CD8A/CD8B receptors on CD8-HAVCR2 cells in metastatic samples. Furthermore, specifically within MBM samples, MBMATCs uniquely engaged CD8-HAVCR2 cells via LAMA4 and FN1 binding to the CD44 receptor. These ligand-receptor pairs suggest a potential immune evasion mechanism: sustained antigen exposure may collectively foster an immunosuppressive microenvironment in both extracranial and brain metastases, while within MBMs, MBMATCs may directly interact with and potentially suppress exhausted T cells through FN1/LAMA4 secretion, concurrently remodeling the brain niche to facilitate tumor cell colonization.

The derivation of the MBM-Index from MBMATCs signatures provided robust prognostic value, independently predicting poorer overall survival in TCGA-SKCM and validation cohorts. It exhibited no significant association with conventional TNM staging, highlighting its potential as an independent biomarker for predicting the risk of brain metastasis in melanoma.

Several limitations warrant consideration. First, sc/snRNA-seq captures a “snapshot” of MBM; longitudinal tracking of MBMATCs evolution may be needed. Second, the impact of prior therapies on the TME in metastatic samples, though minimized by including treatment-naïve samples where possible, could confound microenvironmental comparisons. Third, while the MBM-Index was validated using overall survival (OS) as the primary endpoint in discovery and validation cohorts, future studies should incorporate time-to-brain-metastasis (TBM) as a direct association variable in MBM patients. This refinement would more precisely align the MBM-Index with its biological function as a predictor of brain metastasis risk, enhancing its clinical utility for early intervention strategies. Fourth, rigorous experimental validation is essential to definitively establish the functional properties of MBMATCs and their specific role in driving brain metastasis. Crucially, in vivo studies are required to test the efficacy of predicted therapeutic candidates in physiologically relevant models that recapitulate the brain metastatic niche.

## Conclusions

Our integrated single-cell atlas elucidates the dynamic remodeling of melanoma cells and immune landscapes during brain metastasis. We identify MBMATCs as a putative cellular origin of MBM, characterized by specific transcriptomic programs enabling neural niche adaptation. The accompanying T-cell exhaustion and Treg enrichment create an immunosuppressive milieu that likely contributes to therapeutic resistance. The MBM-Index, derived from MBMATC signatures, emerges as a promising biomarker for stratifying patients at high risk of brain metastasis and predicting survival outcomes independently of traditional staging. These insights lay the groundwork for developing early detection strategies and rationally designed combination therapies targeting both metastatic cells and their immunosuppressive microenvironment to improve outcomes for MBM patients.

## Supporting information

S1 Fig scRNA-seq and snRNA-seq profiling of 26 melanoma samples. (A) t-SNE plots depicting the cell origins by seurat clusters (left panel), samples origin (middle panel) and tissue origin (left panel). (B-C) Boxplot showing the proportions of melanoma cells, T/NK cells and macrophages across PT, ECM and MBM. Statistical difference was calculated by the Wilcoxon rank-sum test.
(TIF)

S2 Fig Immune cells profiling in PTs, ECMs and MBMs. (A-C) t-SNE plots depicting the immune cell origins by seurat clusters (left panel), samples origin (middle panel) and tissue origin (left panel). (D) Dot plot showing percent expression and average expression of labeled macrophage clusters, including four main subtypes. Cluster-specific subtype labels are indicated at the top. (E) Dot plot showing percent expression and average expression of labeled T cell clusters, including nine main functional subsets. Cluster-specific functional labels are displayed at the top.
(TIF)

S3 Fig Pseudotime trajectory analysis of melanoma clusters Mela4 and Mela5. (A-B) Pseudotime trajectory analysis of Mela4 and Mela5 cells, ordered and annotated by pseudotime (left panel), five cellular states (middle panel), three tissues (middle panel) and cell proportions (right panel).
(TIF)

S4 Fig Interaction between MBMATCs and the immune microenvironment. (A) Scatter plot depicting the outgoing and incoming interaction strength for each cell subpopulation within PTs, ECMs and MBMs. (B) Dot plot showing ligand-receptor interactions from specified subpopulations (Mela3, CD8-LEF1, CD8-NKG7, CD8-HIF1A) to the CD8-HAVCR2 subpopulation across PTs, ECMs, and MBMs. Dot size corresponds to the statistical significance (P-value), and color intensity represents the interaction strength.
(TIF)

S5 Fig The MBM-Index showed no significant correlation with clinicopathological features. (A-F) Associations between BM-Index and clinicopathological features in the TCGA-SKCM cohort. Statistical difference was calculated by the Kruskal-Wallis test. T, N, M refer to the AJCC TNM staging system classification (Tumor size, Node involvement, distant Metastasis).
(TIF)

S6 Fig The enriched pathway by all transcripts differentially expressed between MBMATCs and other tumor cells, and between MBM-High and MBM-Low groups. (TIF)

S7 Fig Identification of therapeutic drugs with high drug sensitivity for brain metastasis-associated tumor cells (MBMATCs) and high-brain metastasis (MBM) index patients. (A-I) Differential drug-response analysis (boxplots) and Spearman’s correlation analysis (right panel) for Mirin_1048, PR-4708671_1129 and XAV939_1268.
(TIF)

S1 Table Samples & cell type composition in sc/snRNA-seq. (XLSX)

S2 Table AXL and MITF signature genes. (XLSX)

S3 Table Marker genes in melanoma tumor cells across PT, ECM and MBM by sc/snRNA-seq. (XLSX)

S4 Table Samples & cell type composition in melanoma tumor cells. (XLSX)
