# Supplementary figures and images for: Single-cell transcriptomics profiling reveals cellular origins and molecular drivers underlying melanoma brain metastasis
Source: PLoS One. 2025 Nov 24;20(11):e0336502. doi: 10.1371/journal.pone.0336502 (PMC12643271; doi:10.1371/journal.pone.0336502)

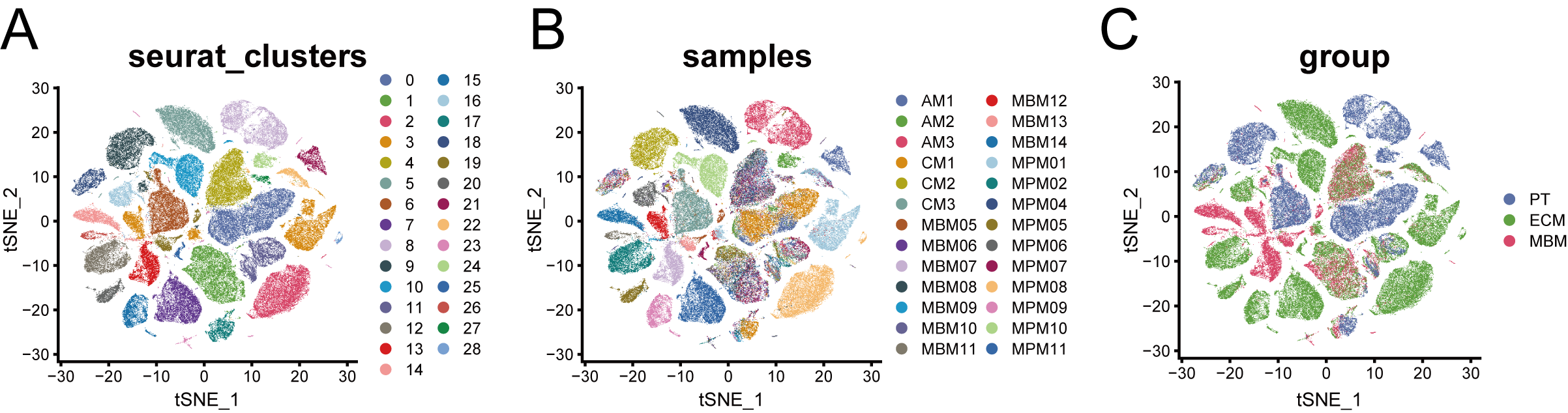

Supplement: S1 Fig — (A) t-SNE plots depicting the cell origins by seurat clusters (left panel), samples origin (middle panel) and tissue origin (left panel). (B-C) Boxplot showing the proportions of melanoma cells, T/NK cells and macrophages across PT, ECM and MBM. Statistical difference was calculated by the Wilcoxon rank-sum test. (TIF) [file pone.0336502.s001.tif]

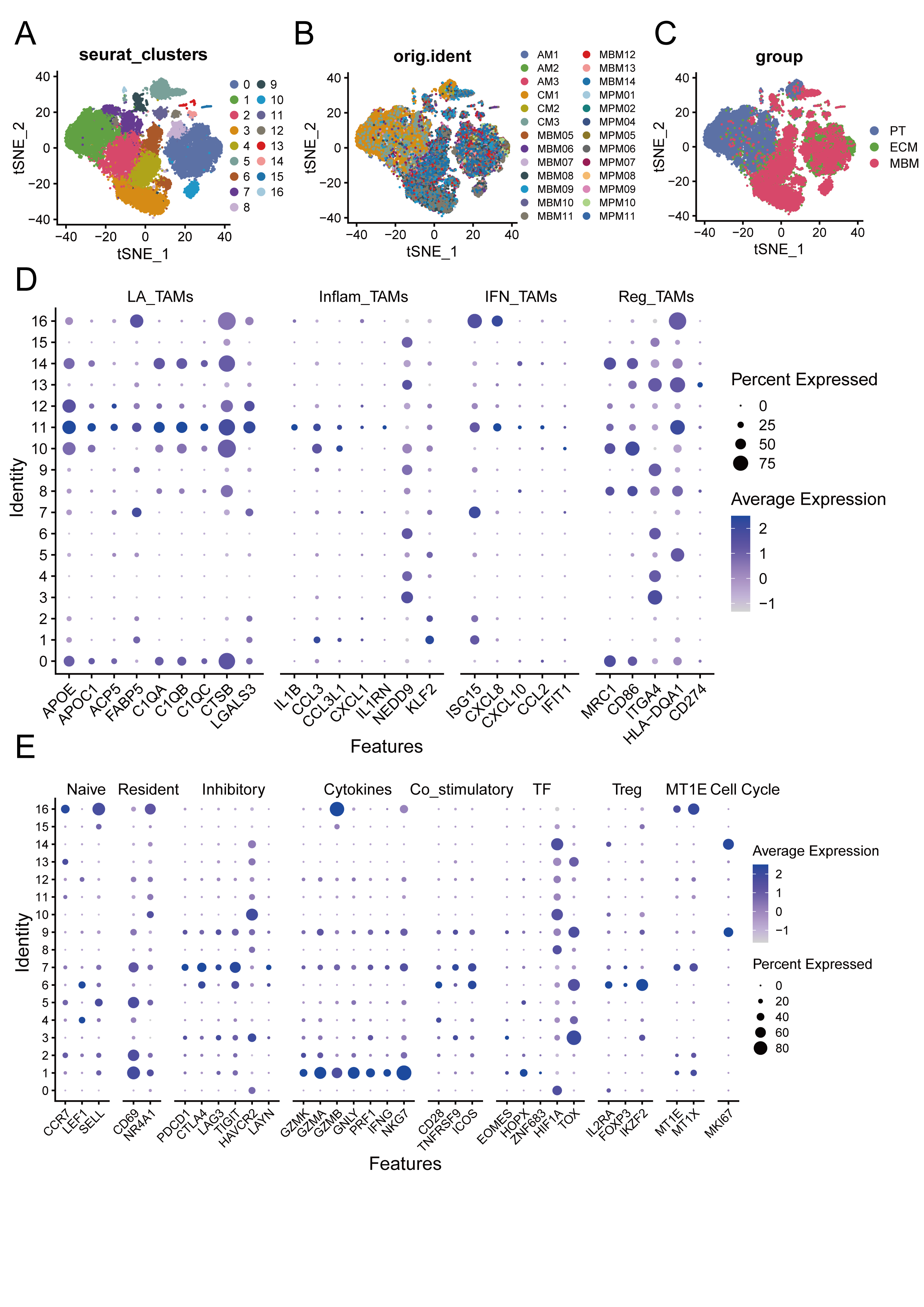

Supplement: S2 Fig — (A-C) t-SNE plots depicting the immune cell origins by seurat clusters (left panel), samples origin (middle panel) and tissue origin (left panel). (D) Dot plot showing percent expression and average expression of labeled macrophage clusters, including four main subtypes. Cluster-specific subtype labels are indicated at the top. (E) Dot plot showing percent expression and average expression of labeled T cell clusters, including nine main functional subsets. Cluster-specific functional labels are displayed at the top. (TIF) [file pone.0336502.s002.tif]

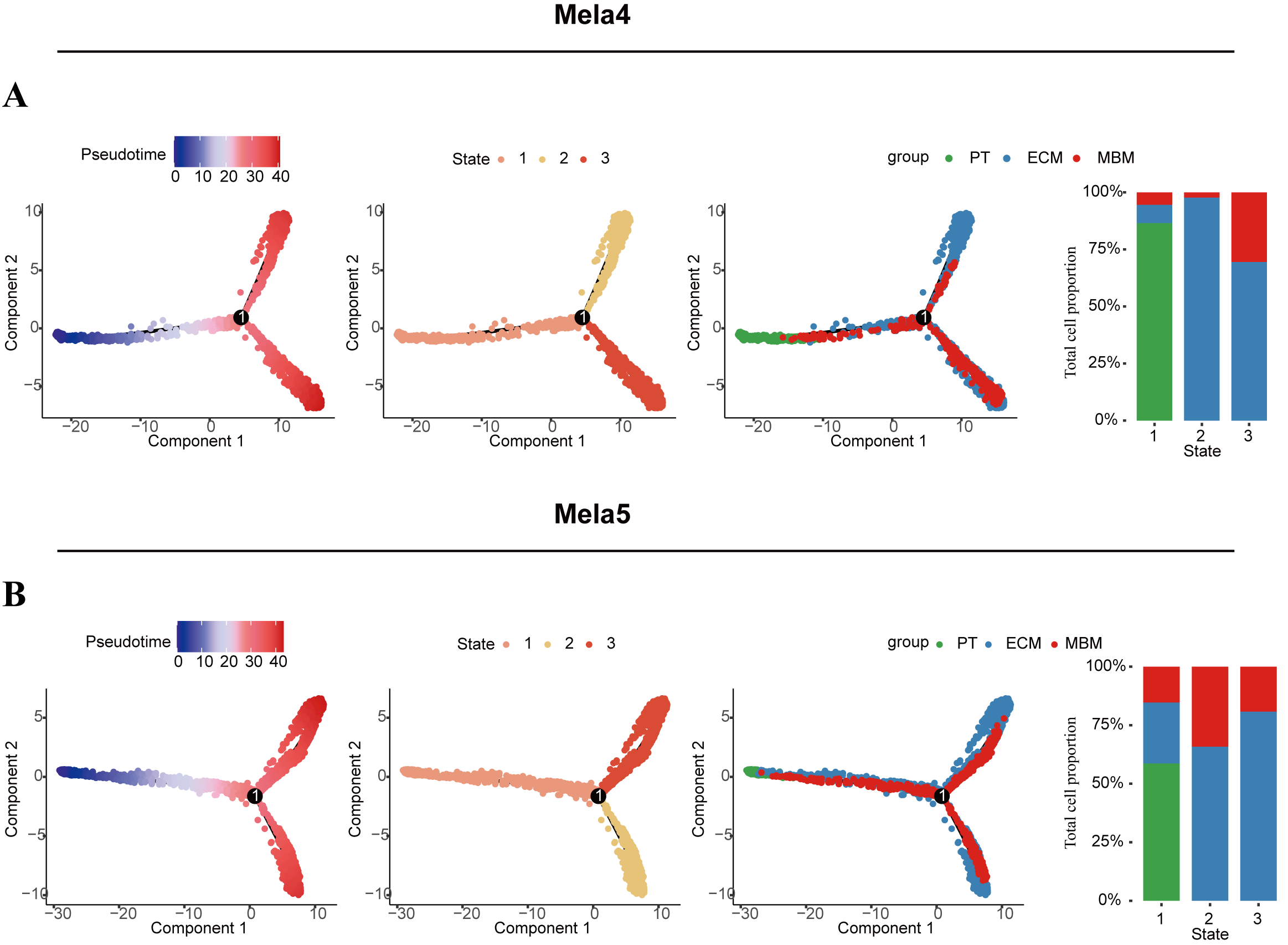

Supplement: S3 Fig — (A-B) Pseudotime trajectory analysis of Mela4 and Mela5 cells, ordered and annotated by pseudotime (left panel), five cellular states (middle panel), three tissues (middle panel) and cell proportions (right panel). (TIF) [file pone.0336502.s003.tif]

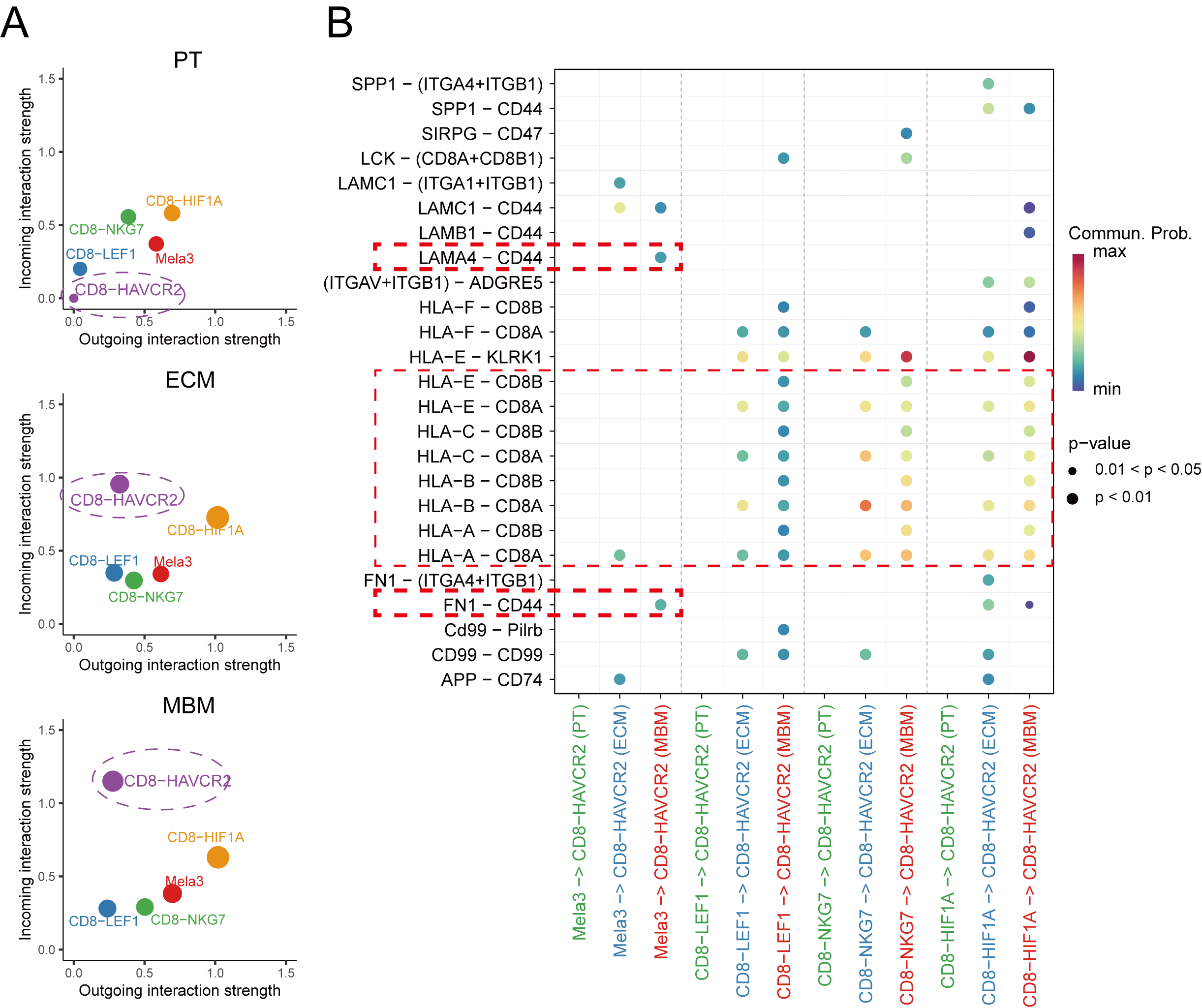

Supplement: S4 Fig — (A) Scatter plot depicting the outgoing and incoming interaction strength for each cell subpopulation within PTs, ECMs and MBMs. (B) Dot plot showing ligand-receptor interactions from specified subpopulations (Mela3, CD8-LEF1, CD8-NKG7, CD8-HIF1A) to the CD8-HAVCR2 subpopulation across PTs, ECMs, and MBMs. Dot size corresponds to the statistical significance (P-value), and color intensity represents the interaction strength. (TIF) [file pone.0336502.s004.tif]

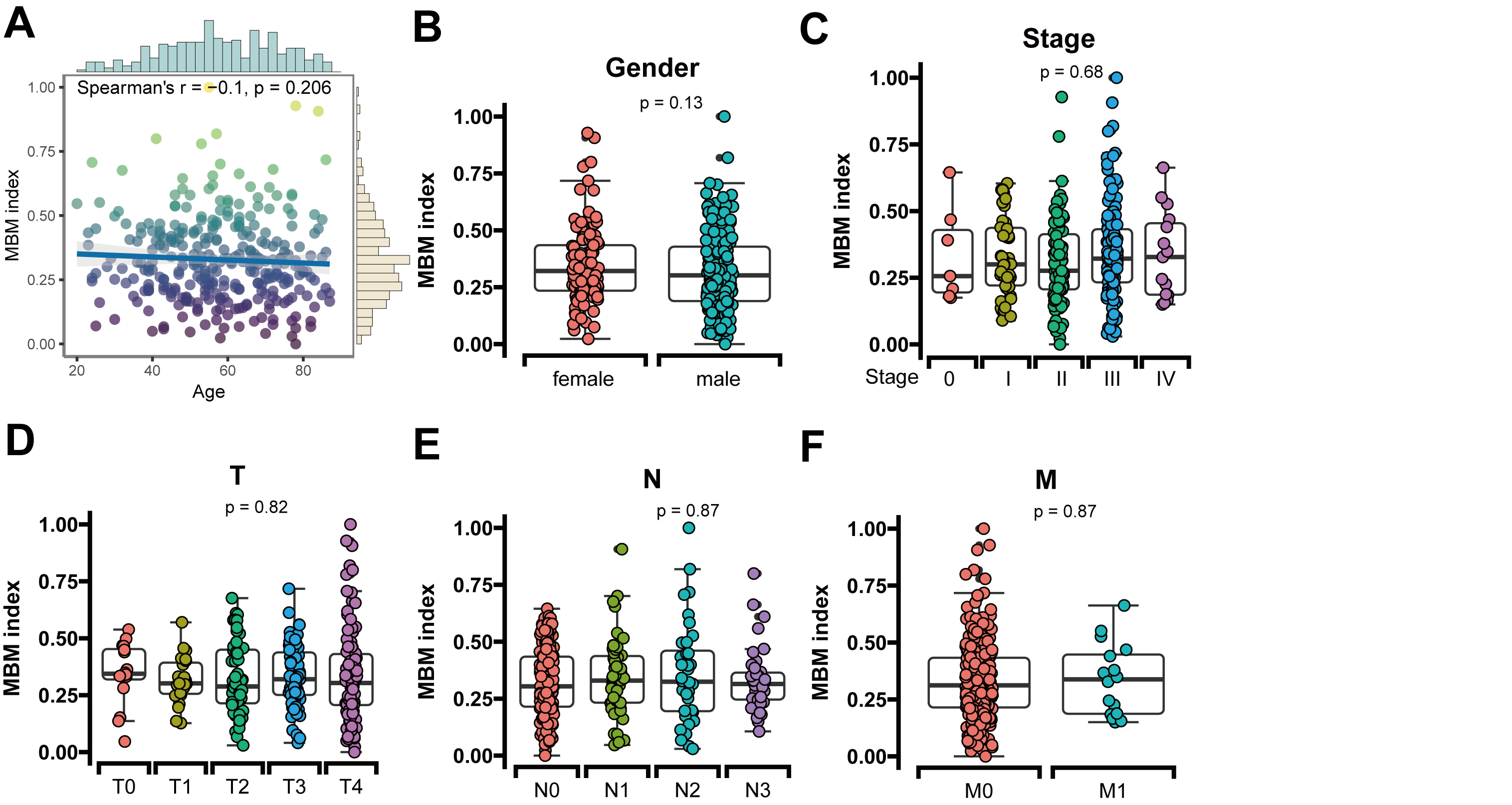

Supplement: S5 Fig — (A-F) Associations between BM-Index and clinicopathological features in the TCGA-SKCM cohort. Statistical difference was calculated by the Kruskal-Wallis test. T, N, M refer to the AJCC TNM staging system classification (Tumor size, Node involvement, distant Metastasis). (TIF) [file pone.0336502.s005.tif]

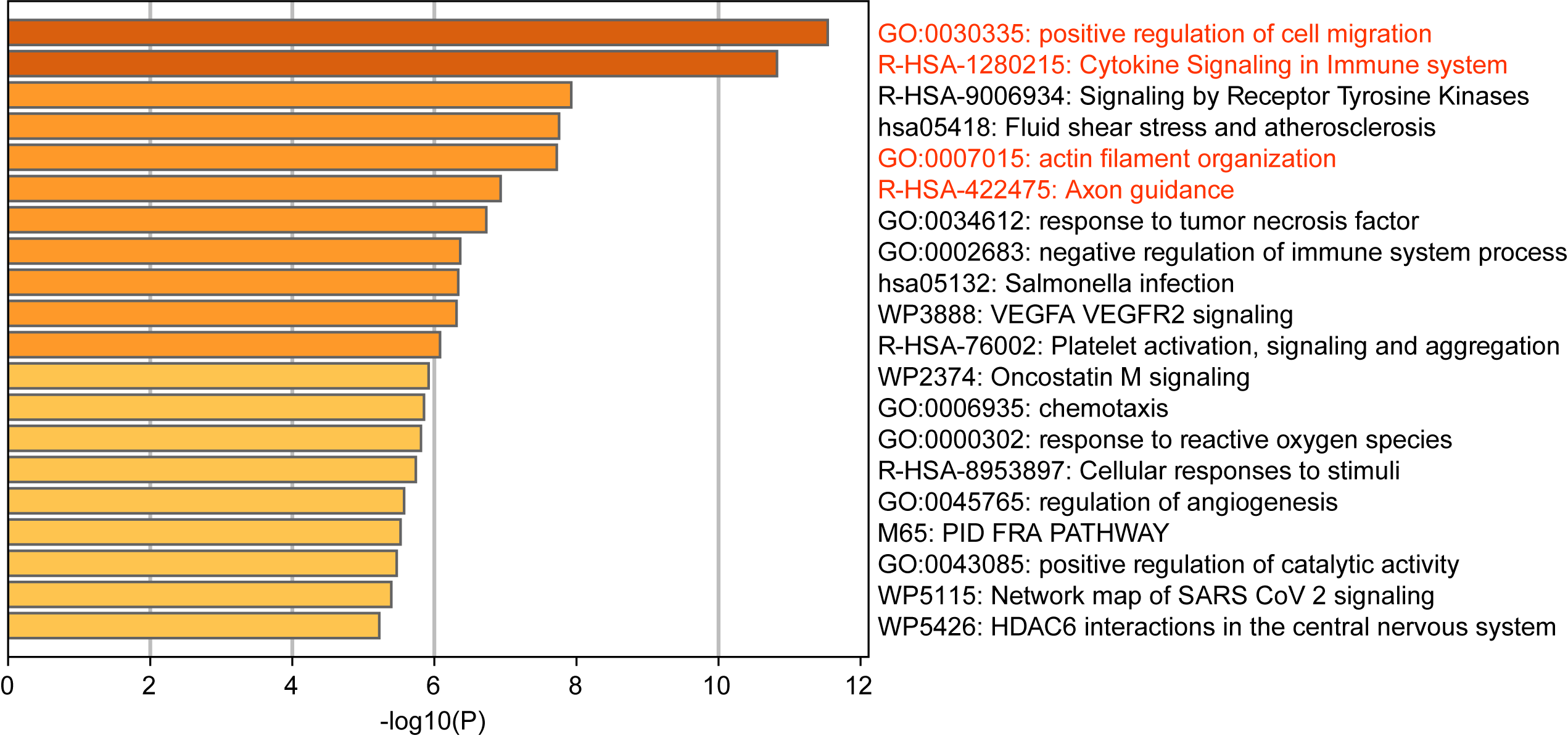

Supplement: S6 Fig — (TIF) [file pone.0336502.s006.tif]

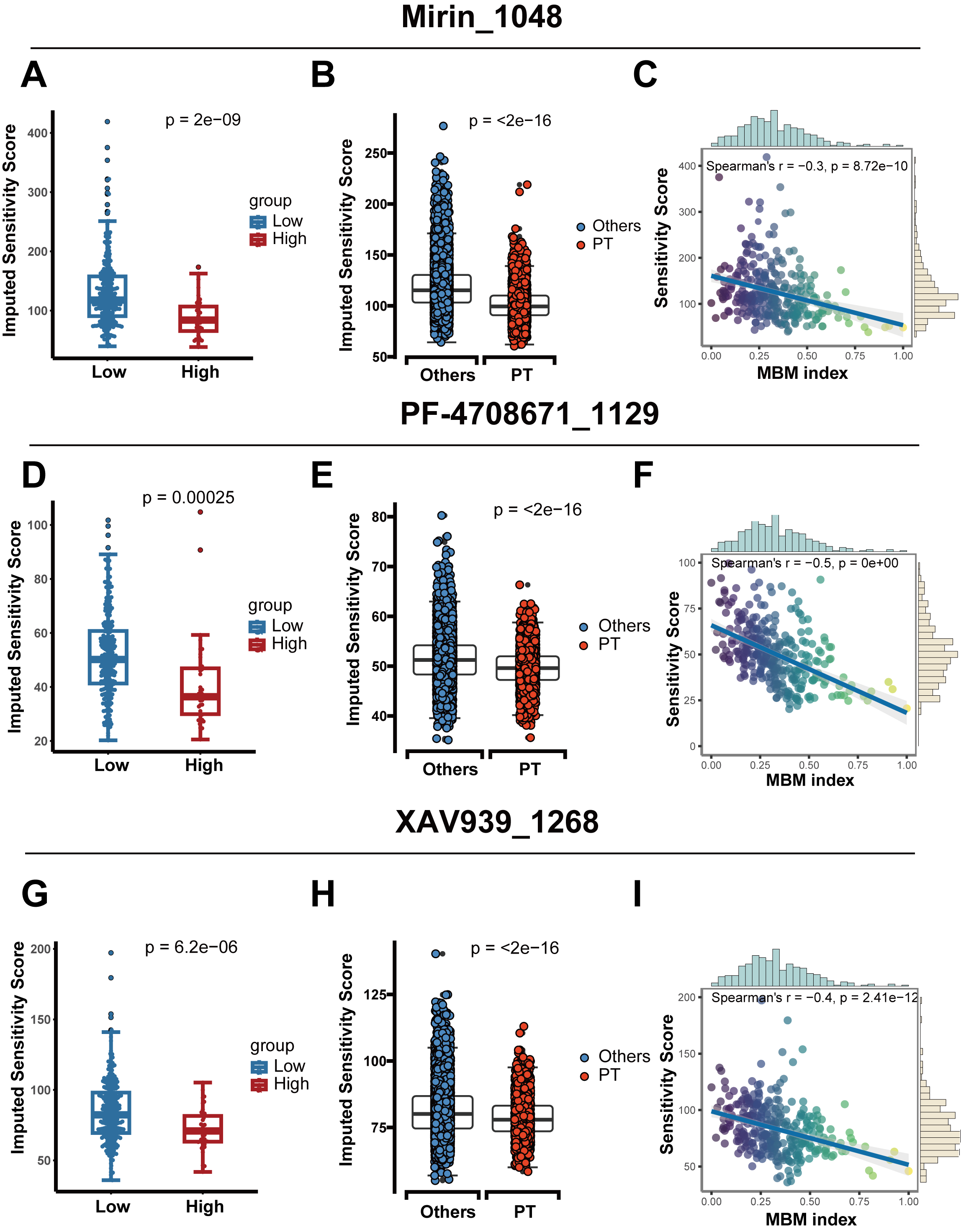

Supplement: S7 Fig — (A-I) Differential drug-response analysis (boxplots) and Spearman’s correlation analysis (right panel) for Mirin_1048, PR-4708671_1129 and XAV939_1268. (TIF) [file pone.0336502.s007.tif]
